# Increasing the uptake of prevention of mother-to-child transmission of HIV services in a resource-limited setting

**DOI:** 10.1186/1472-6963-10-29

**Published:** 2010-01-28

**Authors:** Kwasi Torpey, Mushota Kabaso, Prisca Kasonde, Rebecca Dirks, Maxmillian Bweupe, Catherine Thompson, Ya Diul Mukadi

**Affiliations:** 1 Family Health International/Zambia Prevention, Care, and Treatment Partnership, Lusaka, Zambia; 2 Family Health International/Arlington, VA, USA; 3 Ministry of Health/Zambia, Lusaka, Zambia

## Abstract

**Background:**

As in other resource limited settings, the Ministry of Health in Zambia is challenged to make affordable and acceptable PMTCT interventions accessible and available. With a 14.3% HIV prevalence, the MOH estimates over one million people are HIV positive in Zambia. Approximately 500,000 children are born annually in Zambia and 40,000 acquire the infection vertically each year if no intervention is offered. This study sought to review uptake of prevention of mother-to-child (PMTCT) services in a resource-limited setting following the introduction of context-specific interventions.

**Methods:**

Interventions to improve PMTCT uptake were introduced into 38 sites providing PMTCT services in Zambia in July 2005. Baseline and follow up service data were collected on a monthly basis through September 2008. Data was checked for internal and external consistency using logic built into databases used for data management. Data audits were conducted to determine accuracy and reliability. Trends were analyzed pre- and post- intervention.

**Results:**

Uptake among pregnant women increased across the 13 quarters (39 months) of observation, particularly in the case of acceptance of counseling and HIV testing from 45% to 90% (p value = 0.00) in the first year and 99% by year 3 (p value = 0.00). Receipt of complete course of antiretroviral (ARV) prophylaxis increased from 29% to 66% (p = 0.00) in the first year and 97% by year 3 (p value = 0.00). There was also significant improvement in the percentage of HIV positive pregnant women referred for clinical care.

**Conclusions:**

Uptake of PMTCT services in resource-limited settings can be improved by utilizing innovative alternatives to mitigate the effects of human resource shortage such as by providing technical assistance and mentorship beyond regular training courses, integrating PMTCT services into existing maternal and child health structures, addressing information gaps, mobilizing traditional and opinion leaders and building strong relationships with the government. These health system based approaches provide a sustainable improvement in the capacity and uptake of services.

## Background

Effective interventions for prevention of mother-to-child transmission (PMTCT) were discovered in the late 1990s, yet mother-to-child transmission (MTCT) remains the most significant route of HIV infection among children [1]. With the effectiveness of PMTCT interventions well established, there is an urgent need to scale-up PMTCT programs, particularly in resource-limited settings.

MTCT transmission rates in the United States and Europe are below 2% due to wide coverage and provision of highly effective ART regimen [2,3]. In contrast to the developed world, only 45% of HIV-positive women in low and middle income countries received antiretroviral (ARV) prophylaxis for PMTCT in 2008 [1].

As in other resource-limited settings, the Ministry of Health (MoH) in Zambia is challenged to make affordable and acceptable PMTCT interventions accessible and available. With a 14.3% HIV prevalence, the MOH estimates over one million people are HIV positive in Zambia [4]. Approximately 500,000 children are born annually in Zambia and 40,000 acquire the infection vertically each year if there is no intervention [5].

The steps involved in the provision of core PMTCT programs are often referred to as a cascade. The steps include 1) utilization of antenatal care; 2) receiving pre-test counseling; 3) acceptance of HIV test; and 4) receiving HIV test results & post-test counseling. For seropositive mothers the cascade continues with 5) use of ARV prophylaxis for mother and/or baby; 6) use of labor & delivery services which include specific PMTCT interventions during these periods; and 7) postnatal follow-up of the mother and baby with HIV testing of the exposed infant/child and linkage to care and support. As the term "cascade" suggests, uptake typically decreases at each successive step, which occur as a result of economic, educational, political, social, cultural, and/or health system factors. Ideally, the number of pregnant women seen in the antenatal clinic would be equal to the number of women receiving counseling, testing and received results. Furthermore, the number of pregnant women who test positive for HIV should equal to the number offered a complete course of prophylactic ARVs and also referred for clinic care.

This descriptive review describes the approaches that were used to increase the ability of the health system to increase uptake and retain pregnant women from the point of enrollment into antenatal care through labor and delivery.

### Program Description

In May 2005, Family Health International's (FHI) Zambia Prevention, Care, and Treatment Partnership (ZPCT), was launched in order to strengthen and expand existing HIV/AIDS services in five provinces. The partnership is a six year cooperative agreement funded by the U.S. President's Emergency Plan for AIDS Relief (PEPFAR) through United States Agency for International Development (USAID); it works in collaboration with the Zambian Ministry of Health (MoH) in five of Zambia's nine provinces: Northern, Luapula, Copperbelt, Central, and North-Western. With the exception of the Copperbelt, the provinces selected have the highest poverty index in Zambia [6]. Within these provinces, ZPCT is the primary PEPFAR partner in Zambia. ZPCT's key role is to build capacity in the health care system, allowing for improved access and uptake to quality HIV services, particularly for those most in need. The project supports implementation and scaling up of HIV services by the MOH through training, mentorship, structural refurbishments, strengthening of monitoring and evaluation systems, and provision of equipment and medical supplies. This approach addresses many of the issues of health systems strengthening.

In the first phase, from May 2005 to October 2005, HIV related care and treatment services namely counseling and testing (CT), clinical care, antiretroviral therapy (ART) and PMTCT were initiated in 43 facilities. Out of these 38 offered PMTCT services. The remaining five facilities did not offer PMTCT because they did not have at least two trained healthcare workers as required by national guidelines [5]. In each phase, a needs assessment for each facility was conducted in conjunction with the local District Health Management Team (DHMT) and the Provincial Health Office (PHO). Activities conducted by ZPCT included training health care workers in counseling and testing, PMTCT and commodity management, and intensive onsite mentorship to ensure the confidence and proficiency of the health care workers (HCWs). In addition, training and mentorship in data management was also provided. Job aids for HCWs were developed and existing guidelines were reprinted and distributed to all facilities.

Simultaneously, facilities were refurbished to provide adequate space for service provision. Refurbishment also included procurement of laboratory and other minor medical equipment as well as the creation of testing corners.

In an effort to improve PMTCT uptake, ZPCT employed a variety of strategies to:

• Expand access;

• Provide same day routine HIV testing with opt-out strategy;

• Build human resource capacity;

• Improve facility capacity;

• Foster understanding and reduce stigma;

• Increase male involvement;

• Ensure confidentiality;

• Conduct postnatal follow-up of mother-baby pairs; and

• Improve commodity management.

#### Expand access

To ensure equity of access even for those in the most rural areas, ZPCT implemented a universal access approach. As part of this approach, PMTCT services were integrated at all levels of health care, including tertiary hospitals, provincial, district, primary and community health care settings. Additionally, ZPCT broadened PMTCT service entry points by providing them in antenatal care (ANC) facilities, labor and delivery wards, maternal, newborn and child health clinics, and family planning and STI clinics.

In Zambia, about 90% of pregnant women will attend antenatal clinic at least once, however less than 50% return to deliver in the health facility [7]. In many remote areas, access to ANC and other services is hampered by distance. To address that issue, ZPCT supported an outreach ANC/PMTCT strategy, whereby health care workers (HCWs) from a particular health facility provided outreach ANC services in selected points in the community where distance to the facility was a problem. Schedules were made to rotate the services within the catchment area of the health facility so that each of such outreach points was visited at least once a month. ANC as well as under-five clinic services were provided.

#### Provide same day opt -out HIV testing

The 'opt-out' model of HIV testing provides all antenatal care attendees with counseling for HIV testing, with the option to refuse HIV testing. Multiple studies have supported the approach of routine testing (opt-out) for increased uptake of HIV testing among pregnant women [8-10]. Even though the 'opt out' strategy for HIV testing was a national policy for a number of years in Zambia, it was not operational in facilities. In ZPCT's effort to further increase PMTCT service delivery points, counseling and testing within ANC and labor and delivery rooms were made routine using the 'opt out' strategy. Through training of HCWs and lay providers, counseling and testing is offered to all women who enroll into the ANC as part of routine care except those who decline. This approach was implemented according to national policy in all the supported facilities.

#### Build human resource capacity

Human resource shortages negatively impacted implementation of PMTCT services at every step. Initiation and strengthening services with "opt out" HIV testing with same day results further increased the need for human resources. ZPCT employed a combination of strategies to build human resource capacity by increasing the number of trained HCWs, improving the skills of existing staff and shifting certain tasks to lay providers. The program did not hire new health care workers but used existing Ministry of Health staff. Non-health care workers (i.e. community motivators and lay counselors) were trained to support and provide some PMTCT services. Non-HCWs were used to provide group motivational talks, which shortened the time required for one-on-one pre-test counseling with trained HCWs. Additionally, some non-HCWs were trained in HIV testing and conducted the tests themselves. ZPCT has also trained people living with HIV/AIDS (PLHWA) and lay counselors to provide psychosocial support, make referrals, and help patients adhere to ART.

ZPCT trained and mentored nurses, midwives, doctors and other non-laboratory staff in PMTCT, including rapid HIV testing within the PMTCT context. ZPCT also supported off duty shifts, in which HCWs trained in PMTCT are allowed to work an 'extra shift' and are provided with a transportation reimbursement of US$7 per shift as an incentive which is about 3% of the average monthly salary for nurses [11]. Typically, a nurse may perform 2-4 extra shifts per month.

#### Improve facility capacity

Testing corners were established by ZPCT in May 2005 when it was identified that over 20% of clients did not return for their HIV results [12]. HIV testing corners were designated in rooms where counseling and PMTCT was offered and included a table or desk acting as a laboratory bench, a chair, HIV testing kits, disposable gloves, blood collection instruments and a hand-washing station. In order to facilitate the provision of same day results, testing corners were operated by HCWs and lay providers who conducted rapid HIV testing under the supervision of laboratory technicians for quality control.

For sites where it was not possible to assess the immunological status of clients due to a lack of laboratory facilities, ZPCT implemented a laboratory specimen referral system, in which blood samples were sent to referral laboratories for testing instead of clients traveling long distances to testing facilities. Referral sites house hematology, chemistry and CD4 machines. Specific clinic days were designated for blood drawing and specimen referral to facilities providing ART using motorcycles. Test results were sent back to the centers for patient management within a turn around time of one week.

#### Foster understanding, reduce stigma and involve male partners

A lack of understanding by health care providers and communities are a major barrier to the uptake of HIV testing and use of ARV prophylaxis for the mother and/or baby. Lack of information and misinformation perpetuate stigma, discrimination, and reluctance to take ARV prophylaxis. Uptake of ARV prophylaxis can be increased through health education on MTCT and PMTCT interventions and treatment of HIV/AIDS, as well as through sensitization on the need to deliver at a health facility [13,14].

In order to improve HIV and PMTCT awareness, ZPCT provided health education at community and facility levels with a focus on information programs to improve the knowledge among both women and men regarding PMTCT services. In all the catchment populations surrounding the health facilities, community mobilization and education has been ongoing under the auspices of Neighborhood Health Committees and community based organizations. Mobilization is intense in the beginning and tapers off when the target population has been reached. To specifically increase the acceptability of HIV testing among pregnant women and reduce stigma, traditional and religious leaders were engaged in community sensitization and mobilization. In Luapula province in particular, HCWs encouraged the involvement of traditional leaders within the community. PMTCT providers from the local hospital first led a series of information campaigns targeting traditional and other opinion leaders. Subsequent community meetings were then led by traditional leaders where all males were encouraged to accompany their spouses for their first ANC clinic and be actively involved in their care.

In addition, ZPCT, in partnership with Health Communication Partnership (HCP), worked with male peer groups to mobilize men to participate in ANC and reproductive health (RH) services. Pregnant women who come with their spouses were seen first by the midwife or nurse to promote male involvement in maternal and child health services and PMTCT.

#### Ensure confidentiality

Counseling and testing services require a counseling space that ensures auditory and visual privacy and an area for HIV testing and waste disposal. ZPCT often found inadequate space or no auditory and/or visual privacy available in the facilities. Therefore, whenever possible, ZPCT renovated the facilities to address these inadequacies, which included partitioning or extension of existing rooms to create added private space.

#### Conduct postnatal follow-up of mother-baby pairs

ZPCT's approach placed a clear emphasis on postnatal follow-up of mother-baby pairs. This step has proved to be necessary in increasing uptake of referrals to clinical care and support for both babies and mothers. Mother-baby pairs were followed up in the maternal and child health (MCH) setting and aided through the development of new tools, such as the Mother-Baby Tracking Tool; HIV exposed babies were initiated on co-trimoxazole prophylaxis. Where accessible, babies were tested at six weeks through polymerase chain reaction (PCR). Where PCR was not available, HIV exposed babies were tested at nine and eighteen months. Babies who tested positive for HIV were linked to care and treatment services and the mother supported to provide appropriate feeding options through infant feeding counseling. Exclusive breast feeding was encouraged up to the first six months. Whilst formula for infant feeding was not offered, women who opted not to breast feed were counseled to ensure replacement feeding was acceptable, feasible, affordable, safe and sustainable.

#### Commodity management

Initially there were commodity stock outs of HIV test kits and supplies. Stock outs resulted in interruptions in service provision. ZPCT supported training for HCWs in commodity and data management to facilitate correct and timely orders.

## Methods

The data was collected from 38 sites providing PMTCT services in five provinces of Zambia by July 2005. This was then followed by the introduction of a set of interventions to improve PMTCT uptake. Baseline and follow up data was collected as part routine service statistics compiled monthly through September 2008. Collected data was checked for internal and external consistency using logic built into the databases that are used for the data management. Additionally, data audits were conducted on a quarterly basis to determine the accuracy and reliability of the already reported data. Trends were analyzed pre- and post- intervention to assess the effect of interventions.

Approval: Data was approved for use by Ministry of Health and Zambia Prevention Care and Treatment Partnership

## Results

**Figure 1:**
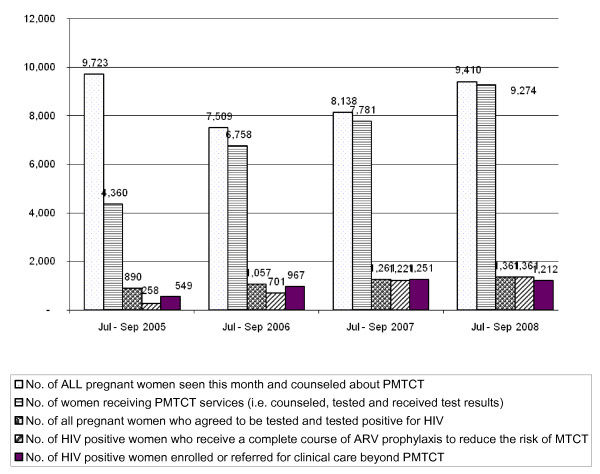
**PMTCT Cascade during 3 Years of Implementation, 2005 to 2008 [n = 38 health facilities]**.

**Figure 2:**
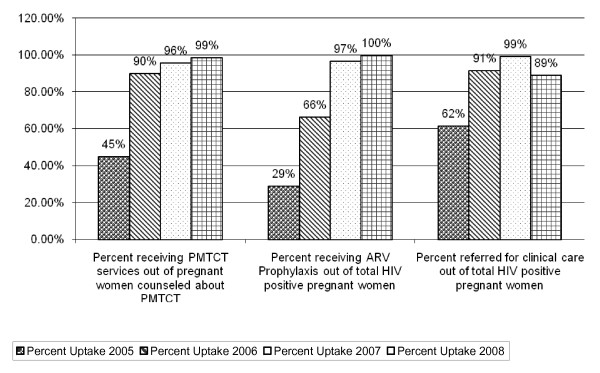
**PMTCT Cascade of uptake during 3 Years of Implementation, 2005 to 2008 [n = 38 health facilities]**.

**Figure 3:**
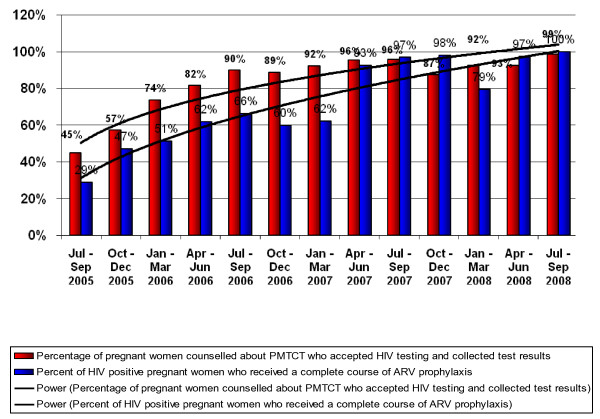
**Uptake of Select PMTCT Services, July 2005 to Sep 2008 (n = 38 ZPCT health facilities)**.

After three years of implementation, substantial increases were observed in the proportion and number of women accessing PMTCT services (see Figures 1, 2, 3).

At baseline (July 2005 to September 2005), 9723 pregnant women were counseled about PMTCT, 4630 (45%) were tested for HIV and received their results, 890 tested positive for HIV, 549 seropositive pregnant women were enrolled or referred to clinical care beyond PMTCT, and 258 seropositive pregnant women (29%) received a complete course of ARV prophylaxis. One year later, from July 2006 to September 2006, 7509 pregnant women were counseled about PMTCT, 6758 (90%) (p value = 0.00) were tested for HIV and received their results, 1057 tested positive for HIV, 967 seropositive pregnant women were enrolled or referred to clinical care beyond PMTCT, and 701 seropositive pregnant women received a complete course of ARV prophylaxis (66%) (p value = 0.00). The trend is sustained in year 2 and 3 with percentage of pregnant women counseled, tested and receiving results increasing to 99% with those receiving complete course of prophylaxis reaching 100% (see Figures 1, 2, 3).

## Discussion and Conclusions

By using a participatory approach, the program has been successful in increasing the uptake of PMTCT services throughout the cascade. In spite of limited resources, uptake had clearly increased in the 38 sites since the introduction of the interventions, particularly in the case of acceptance of counseling and HIV testing, receipt of complete course of ARV prophylaxis, and referral for clinical care beyond PMTCT.

The low proportion of seropositive women who receive a complete course of ARV prophylaxis was a challenge. Although Figures 1 and 2 show a significant increase in the uptake of ARV prophylaxis, from 29% in July - September 2005 to 66% during the same period in 2006, attrition remained an obstacle. The national PMTCT guidelines state that a nevirapine (NVP) tablet should be given to seropositive women at 32 weeks of pregnancy with instructions on when to ingest it once in labor [7,15]. The reality, however, is that over 90% of pregnant women attend a health facility at least once but less that 50% return to deliver their babies [7].

To address this barrier, ZPCT supported sites gave NVP to seropositive pregnant women the first time they visited a health facility, coupled with instructions for its use at home once in labor. Therefore women who did not return to a facility had access to NVP and instructions on its use. In addition, eligible pregnant women were initiated on ART for their own health. ZPCT used this strategy to improve uptake of ARV prophylaxis for seropositive mothers and continues to follow this approach. A study conducted in 2005 reported that 68% of HIV-infected women in Lusaka, Zambia do actually ingest the NVP tablet given to them during an ANC visit [16]. Dispensing NVP to an HIV positive pregnant women does not necessarily translate to ingestion, however, not providing the drug certainly leads to non-ingestion.

Unlike a controlled research setting, implementation of interventions cannot be done one after the other to assess relative contribution of each component in the field. The key indicator is to ensure that most HIV positive women receive prophylaxis to reduce pediatric infection whilst retaining women within the cascade to improve uptake. The most important step is to increase the entry point for all pregnant women to access counseling and testing services thereby having the opportunity to receive prophylaxis, post natal care and other services in the health facility. Capacity building of health care workers and lay providers, opt out approach and providing same testing and results are the most critical interventions that influences uptake in the early steps of the cascade.

A recent comprehensive PMTCT cost report in ZPCT supported facilities showed a cost range of $113 to $126 per mother depending on whether the services are provided in a hospital or health center. The cost element includes clinical labor, drugs and supplies for the delivery of more efficacious regimen from 32 weeks, laboratory tests, administrative and support labor, capital costs of building and equipments, and other expenditures like operating costs, district and project support [17]. This compares very favorably with other cost studies [18,19].

ZPCT's experience demonstrates how innovative and context-appropriate strategies can effectively increase uptake of PMTCT services - even in resource-limited settings. Strategies which take into account health systems strengthening are more likely to show durable and sustained results over a long period. Several key lessons may be learned from the approaches ZPCT used to improve uptake of PMTCT.

First, scale up of services is possible even with limited human resources but requires innovative alternatives such as multitasking, improving the patient flow, and incorporating non-health workers including supporting off duty shifts for existing staff to provide additional coverage for health facilities. Furthermore, the use of other non-laboratory workers (both health and non-health workers) allowed same day HIV test results possible in places with limited or no laboratory staff and reduces waiting times. In addition, the use of lay counselors to conduct counseling and testing to complement the efforts of health care workers has contributed to improved uptake. However, these efforts require changes to policy and regulatory framework to make it feasible.

Technical assistance, particularly hands-on mentorship, is necessary beyond the regular training courses for services to be implemented well, especially with operationalizing routine HIV testing and ensuring same day results.

Next, expanded PMTCT services should be integrated into the existing maternal and child health structure, which requires strengthening the health system management infrastructure and training and mentoring of current health care workers.

Information gaps related to PMTCT exist and must be addressed. Misinformation leads to stigmatization, discrimination, and low services uptake. Subsequently, male partners should become integrated into the PMTCT process. Traditional leaders/opinion leaders play an important role in mobilizing communities to access HIV/AIDS services and specifically to increase male involvement in PMTCT services.

Finally, a strong relationship with the government at all the levels of the health system is critical. PMTCT programs planning to integrate expand or rapidly scale up must have government support and ownership.

## Competing interests

The authors declare that they have no competing interests.

## Authors' contributions

KT, MK and PK conceived the study, participated in the design and helped draft the manuscript. RD, MB, CT, YDM participated in the design and helped draft the manuscript. MK and RD did the statistical analysis. All authors read and approved the final manuscript.

## Pre-publication history

The pre-publication history for this paper can be accessed here:

http://www.biomedcentral.com/1472-6963/10/29/prepub

## References

[B1] WHO/UNAIDS/UNICEF Towards Universal Access: Scaling up HIV priority interventions in the health sector Progress Report 2009

[B2] Mofenson LM "Successes and challenges in the perinatal HIV-1 epidemic in the United States as illustrated by the HIV-1 serosurvey of childbearing women." Archives of Pediatric & Adolescent Medicine 2004 158 4 422 425 10.1001/archpedi.158.5.422 15123471

[B3] European Collaborative Study "Mother-to-child transmission of HIV in the era of highly active antiretroviral therapy." Clinical Infectious Diseases 2005 40 3 458 465 10.1086/427287 15668871

[B4] Central Statistical Office Zambia/Macro International Zambia Demographic and Health Survey 2007 Key Findings 2009 Calverton, Maryland, USA: CSO and Macro International Inc

[B5] Ministry of Health (MOH) [Zambia] PMTCT National Protocol Guidelines 2008 Lusaka, Zambia: MOH

[B6] Central Statistical Office (CSO) [Zambia] Living Conditions Monitoring Survey Report 2004 2005 Lusaka, Zambia: Central Statistical Office

[B7] Ministry of Health (MOH) [Zambia] Annual Health Statistics Bulletin 2006 2007 Lusaka, Zambia: MOH

[B8] Anderson J Koenig J Lampe M Wright R Leiss J Saul J "Achieving universal HIV screening in prenatal care in the United States: provider persistence pays off." AIDS Patient Care and STDs 2005 19 4 247 252 10.1089/apc.2005.19.247 15857196

[B9] Homsy J Kalamya JN Obonyo J Ojwang J Mugumya R Opio C Mermin J "Routine intrapartum HIV counseling and testing for prevention of mother-to-child transmission of HIV in a rural Ugandan hospital." Journal of Acquired Immune Deficiency Syndromes 2006 42 2 149 154 10.1097/01.qai.0000225032.52766.c2 16760796

[B10] Perez F Zvandaziva C Engelsmann B Dabis F "Acceptability of routine HIV testing ("Opt-Out") in antenatal services in two rural districts of Zimbabwe." Journal of Acquired Immune Deficiency Syndromes 2006 41 4 514 520 10.1097/01.qai.0000191285.70331.a0 16652062

[B11] Mukasa E The Human Resource Crisis in the Zambian Health sector - a discussion paper Medical Journal of Zambia 2009 35 3 81 87

[B12] Torpey K McGill S Kasonde P Tembo M Sialwiindi N Wigley M "'Testing Corners': A key strategy in supplying same day HIV results in Zambia Prevention, Care and Treatment Partnership (ZPCT) supported provinces of Zambia." XVI International AIDS Conference; Toronto, Canada 2006

[B13] Kiarie JN Kreiss JK Richardson BA John-Stewart GC Compliance with antiretroviral regimens to prevent perinatal HIV-1 transmission in Kenya AIDS 2003 17 1 65 71 10.1097/00002030-200301030-00009 12478070 PMC3387271

[B14] Welty T Bulterys M Welty E Tih P Ndikintum G Nkuoh G Nkfusai J Kayita J Nkengasong J Wilfert C "Integrating prevention of mother-to-child HIV transmission into routine antenatal care; the key to program expansion in Cameroon." Journal of Acquired Immune Deficiency Syndromes 2005 40 4 486 493 10.1097/01.qai.0000163196.36199.89 16280706

[B15] Ministry of Health [Zambia] National Protocol guidelines: Integrated mother to child transmission of HIV/AIDS 2006 Lusaka, Zambia: MOH

[B16] Stringer J Sinkala M Maclean C Levy J Kankasa C DeGroot A Effectiveness of city wide program to prevent mother to child HIV transmission in Lusaka, Zambia AIDS 2005 19 1309 1315 10.1097/01.aids.0000180102.88511.7d 16052086 PMC2745046

[B17] Bratt J Torpey K Gondwe Y Cost of HIV/AIDS Outpatient services delivered through Zambian public health facilities Family Health International, Lusaka, Zambia 2009 10.1111/j.1365-3156.2010.02640.x 20958891

[B18] Desmond C Boyce C Monitoring HIV/AIDS Interventions: Assessing the cost of a rural PMTCT pilot in Eastern Cape Human Sciences Research Council, Cape Town, South Africa 2004

[B19] Dandona L Kumar SG Ramesh YK Rao MC Marseille E Kahn J Outputs, cost and efficiency of public sector centers for PMTCT in Andra Pradesh, India BMC Health Services Research 2008 8 doi 10.1186/1472-6963-8-26 10.1186/1472-6963-8-26 18234117 PMC2267788

